# Inhibition of the miR-155 target NIAM phenocopies the growth promoting effect of miR-155 in B-cell lymphoma

**DOI:** 10.18632/oncotarget.6165

**Published:** 2015-10-19

**Authors:** Izabella Slezak-Prochazka, Joost Kluiver, Debora de Jong, Katarzyna Smigielska-Czepiel, Gertrud Kortman, Melanie Winkle, Bea Rutgers, Jasper Koerts, Lydia Visser, Arjan Diepstra, Bart-Jan Kroesen, Anke van den Berg

**Affiliations:** ^1^ Department of Pathology and Medical Biology, University of Groningen, University Medical Center Groningen, Groningen, The Netherlands; ^2^ Biosystems Group, Institute of Automatic Control, Silesian University of Technology, Gliwice, Poland

**Keywords:** B-cell lymphoma, NIAM, Ago2-IP, miR-155, TBRG1

## Abstract

Several studies have indicated an important role for miR-155 in the pathogenesis of B-cell lymphoma. Highly elevated levels of miR-155 were indeed observed in most B-cell lymphomas with the exception of Burkitt lymphoma (BL). However, the molecular mechanisms that underlie the oncogenic role of miR-155 in B-cell lymphoma are not well understood. To identify the miR-155 targets relevant for B-cell lymphoma, we performed RNA immunoprecipitation of Argonaute 2 in Hodgkin lymphoma (HL) cells upon miR-155 inhibition and in BL cells upon ectopic expression of miR-155. We identified 54 miR-155-specific target genes in BL cells and confirmed miR-155 targeting of *DET1*, *NIAM*, *TRIM32*, *HOMEZ*, *PSIP1* and *JARID2*. Five of these targets are also regulated by endogenous miR-155 in HL cells. Both overexpression of miR-155 and inhibition of expression of the novel miR-155 target gene *NIAM* increased proliferation of BL cells. In primary B-cell lymphoma NIAM-positive cases have significant lower levels of miR-155 as compared to NIAM-negative cases, suggesting that NIAM is also regulated by miR-155 in primary B-cell lymphoma. Thus, our data indicate an oncogenic role for miR-155 in B-cell lymphoma which involves targeting the tumor suppressor NIAM.

## INTRODUCTION

MiRNAs are short (21-23 nucleotides) non-coding RNA molecules that mediate posttranscriptional silencing of their target genes [1]. MiRNA-dependent expression regulation is crucial in cellular processes including cell cycle, apoptosis and proliferation. Deregulated miRNA levels have been observed in various hematological malignancies, including B-cell lymphomas [2].

MiR-155 is one of the most frequently studied miRNAs in normal and malignant B-cells. Studies in mice revealed an important regulatory role of miR-155 in diverse aspects of the immune response including B-cell development. Most germinal center (GC) B cells express the primary transcript of miR-155, *BIC / MIR155HG*, and harbor high levels of the mature miR-155 in the course of the GC response [3]. Several studies implicate an important role for miR-155 in the pathogenesis of B-cell leukemia and lymphoma. *O*verexpression of miR-155 driven by the B-cell-specific E&micro;-enhancer induced pre-B-cell lymphoma in miR-155 transgenic mice [4]. In a similar study induction of miR-155 in murine lymphoid tissues caused a clonal, transplantable pre-B-cell malignancy that was dependent on miR-155 expression [5]. In human, high miR-155 levels were observed in GC B cell-derived lymphomas like Hodgkin lymphoma (HL), chronic lymphocytic leukemia, primary mediastinal B-cell *lymphoma* and diffuse large B-cell lymphoma [6-8]. In contrast, Burkitt lymphoma (BL) is characterized by very low miR-155 levels [9]. Over the past few years several miR-155 target genes involved in functioning of normal hematopoietic cells have been identified [10-12]. However, it is largely unknown which target genes are involved in the pathogenesis of B-cell lymphoma.

To identify miR-155 target genes relevant for the pathogenesis of B-cell lymphoma we performed Ago2-immunoprecipitation in B-cell lymphomas with high and low miR-155 expression levels. Next, we tested which of the identified targets were involved in the oncogenic effect of miR-155 overexpression and showed that downregulation of NIAM reproduced the enhanced growth phenotype of miR-155 overexpression. Finally, we show that miR-155 levels are in general high in NIAM-negative B-cell lymphomas and vice versa, supporting a role for miR-155 mediated downregulation of NIAM in the pathogenesis of B-cell lymphoma.

## RESULTS

### Unbiased genome-wide identification of miR-155 target genes in B-cell lymphoma

To determine the target genes of miR-155 in B-cell lymphoma we performed Ago2-RIP-Chip following two strategies. On one hand we stably overexpressed miR-155 in a BL cell line (ST486) known to have low miR-155 levels. On the other hand we inhibited miR-155 with a miRNA-155 sponge construct in 2 HL cell lines (KM-H2 and L1236), both known to have high miR-155 levels. Overexpression of miR-155 in ST486 cells was confirmed by qRT-PCR and revealed a ∼500 fold increase in miR-155 levels (Suppl. Fig. S1A). These levels are comparable to the endogenous miR-155 levels observed in HL cell lines KM-H2 and L1236. Efficiency of the IP procedure was confirmed by Western blot and miRNA qRT-PCR (Suppl. Fig. S1B-S1D).

We defined the miRNA targetome as all transcripts that were ≥ 2-fold enriched in the immunoprecipitated (IP) fraction compared to the total (T) fraction (IP fold enrichment, IP/T ratio ≥ 2). The number of Ago2-IP-enriched probes was similar in all three cell lines, ranging between 12.5%-17.7% of the consistently-expressed probes (Suppl. Table S1). To identify the miR-155-specific targets in ST486 cells we determined which probes had an IP/T ratio of ≥ 2-fold in miR-155-transduced cells and showed an at least 2-fold lower IP/T ratio in EV-transduced cells. In total 64 probes (3.5% of all IP enriched probes) detecting 54 different genes fulfilled these criteria (3 probes did not correspond to any known gene, Suppl. Table S2). Similar analyses were performed for the two HL cell lines, i.e. probes with a ≥ 2-fold IP enrichment in EV-transduced cells and an at least 2-fold lower IP enrichment in miR-155 sponge-transduced cells. This revealed only 19 (0.5% of all IP probes) and 6 (0.3% of all IP probes) probes that fulfill these criteria in KM-H2 and L1236 cells, respectively (data not shown).

**Figure 1:**
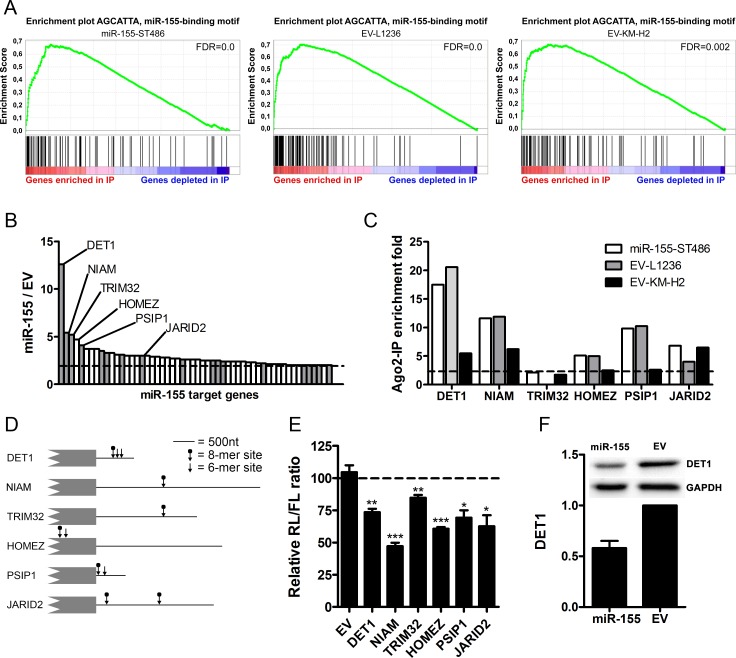
Identification of miR-155 target genes using Ago2-immunoprecipitation **A**. Gene set enrichment plot of the miR-155-binding site motif comparing the Ago2-IP fraction to the total fraction shows strong enrichment of miR-155 target genes in the IP fractions of miR-155-ST486, EV-L1236 and EV-KM-H2 cells. **B**. Overview of the 54 genes that were enriched more prominently in the Ago2-IP fraction of miR-155-ST486 cells compared to that of EV-ST486 cells. The names of the 6 genes selected for further analysis are indicated. Grey bars indicate genes that are predicted miR-155 targets according to TargetScan. The dashed line indicates the cutoff of 2-fold higher enrichment in the Ago2-IP fraction of miR-155-ST486 compared to EV-ST486 cells. The total list of genes is shown in Table S2. **C**. Five of the six selected genes are also enriched in the Ago2-IP fraction of HL cells. Shown are the IP/T (immunoprecipitated fraction/total fraction) ratios of the six selected miR-155 target genes. Five of these genes, *DET1, NIAM, HOMEZ, PSIP1* and *JARID2* also show IP/T>2 in EV-L1236 and EV-KM-H2 cells. *TRIM32* is not expressed in L1236 and 1.75 fold enriched in EV-KM-H2 cells. **D**. Schematic overview of 3′UTR or CDS (*HOMEZ*) regions with the positions of the predicted miR-155 binding sites indicated by the arrows (6-mer and 8-mer sites). For *NIAM* and *PSIP1*, the 3′UTR of the isoforms containing the miR-155 binding sites are shown (ENST00000441174 and NM_021144, respectively). **E**. All 6 selected genes, but not the EV control, are targeted by miR-155 in luciferase reporter assay. For each construct, the Renilla/Firefly luciferase (RL/FL) ratio upon co-transfection with the miR-155 precursor is shown relative to a negative control co-transfection that was set to 100%. P-values were calculated with an unpaired t-test (* *p* < 0.05, ** *p* < 0.01, *** *p* < 0.001). **F**. DET1 protein level was decreased in miR-155-ST486 compared to EV-ST486 cells. Western blot for DET1 relative to GAPDH, EV-ST486 was set as 1, the average of 2 experiments is shown.

Next, we performed gene set enrichment analysis (GSEA) to determine whether specific gene sets were enriched in the IP fractions. We noted that 40-75% of the 20 most enriched gene sets corresponded to miRNA binding site motif gene sets, indicating an efficient enrichment of miRNA target genes in all IP fractions. As a result of the overexpression of miR-155 in ST486 cells, the rank of the miR-155-binding site motif gene set was increased from 46^th^ in EV-ST486 to 13^th^ in miR-155-ST486 cells (Figure 1A) (Suppl. Table S3). In HL cells, the EV-transduced lines already showed clear enrichment of miR-155 target genes in the IP fraction (Figure 1A). However, the ranking of the miR-155-binding site motif set did not decrease clearly in KM-H2 (rank 25^th^ to rank 24^th^) or L1236 (rank 8^th^ to rank 9^th^) cells upon overexpression of the miR-155 sponge (Suppl. Tables S4, S5). Thus, GSEA shows that in ST486 cells miR-155 targets are enriched in the Ago2-IP fraction upon miR-155 overexpression, whereas HL cells do not show a clear depletion of miR-155 targets upon miR-155 inhibition. The GSEA results for the HL cell lines, as well as the more limited number of putative specific miR-155 targets, suggested that the miR-155 sponge did not effectively sequester all miR-155 molecules, probably because of the very high endogenous miR-155 expression levels in KM-H2 and L1236 cells (Fig. S1A).

Analysis of the IP/T ratios of the 54 ST486 miR-155 target genes in the HL cell lines, revealed enrichment for 45 out of 53 (85%) and 25 out of 43 (58%) target genes in EV-transduced L1236 and KM-H2 cells respectively, indicating that these targets are regulated by one or more miRNAs in the wild type HL cell lines (Suppl. Fig. S2). The other ST486 miR-155 targets were either not expressed in L1236 and KM-H2 cells (1 and 11), more mildly enriched (1.5 ≤ IP/T ≤ 2 for 3 and 10) or not enriched (IP/T ≤ 1.5 for 5 and 8).

The enrichment of miR-155 targets in miR-155-ST486 compared to EV-ST486 cells ranged from 2.0 to 12.6 fold (Figure 1B). *In silico* validation indicated that 26 out of 54 genes (48%) contained an 8-mer (AGCATTAA) and 43 genes (80%) a 6-mer (GCATTA) miR-155 binding site in their 3′UTRs (Suppl. Table S2). Moreover, 33% (18 out of 54) of the identified genes were predicted to be miR-155 targets by TargetScan [13]. This is a significant increase as compared to the 1.7% predicted miR-155 targets among all expressed genes (*p* < 0.0001, Chi-square test). Thus, we identified 54 miR-155 target genes by overexpression of miR-155 of which the vast majority was also targeted by miRNAs in B-cell lymphoma cell lines with high endogenous levels of miR-155.

### Validation of miR-155 target genes

We selected six genes identified by Ago2-RIP-Chip in ST486 cells for validation, i.e. one known miR-155 target gene, Jumonji AT rich interactive domain 2 *(JARID2)*, and the five genes with the highest Ago2-IP enrichment upon miR-155 overexpression (>4-fold, Figure 1B, Suppl. Table S2), i.e. De-Etiolated-1 homolog *(DET1)*, Nuclear Interactor of ARF and Mdm2 *(NIAM,* the protein-coding transcript splice variant of the *TBRG1* locus*)*, Tripartite motif-32 *(TRIM32)*, Homeobox leucine zipper *(HOMEZ)* and PC4 and SFRS1 interacting protein 1 *(PSIP1)*. We confirmed Ago2-IP enrichment in the HL EV-transduced cells for 5 of the 6 targets (Figure 1C). The 6^th^ gene, i.e. *TRIM32*, was not expressed in L1236 and was borderline enriched in KM-H2 cells (IP/T ratio = 1.75). For 4 out of the 5 miR-155 targets the IP enrichment was mildly decreased upon miR-155-sponge overexpression in L1236 and for 5 out of 6 in KM-H2 cells. This suggests that these genes are also targeted by endogenously expressed miR-155 in HL cells (Suppl. Fig. S2). To validate a direct interaction between miR-155 and these targets we cloned their predicted miR-155 binding sites into the psiCHECK2 luciferase vector. For *DET1, NIAM*, *TRIM32*, *PSIP1* and *JARID2* these sites were located in their 3′UTRs and for *HOMEZ* they were predicted in the coding sequence (Figure 1D). For all 6 genes, co-transfection of the resulting luciferase constructs with a miR-155 precursor into ST486 wild-type cells resulted in significantly decreased relative luciferase levels (range 15-53%) compared to co-transfection with a negative control precursor (Figure 1E). In addition, the highest Ago2-IP-enriched gene upon miR-155 overexpression in ST486 cells, i.e. *DET1,* showed a decreased protein level upon miR-155 induction in ST486 cells (Figure 1F). Thus, we confirmed that *DET1*, *NIAM*, *TRIM32*, *HOMEZ*, *PSIP1* and *JARID2* are valid miR-155 target genes.

### Both miR-155 overexpression and inhibition of miR-155 target NIAM result in enhanced cell growth

**Figure 2:**
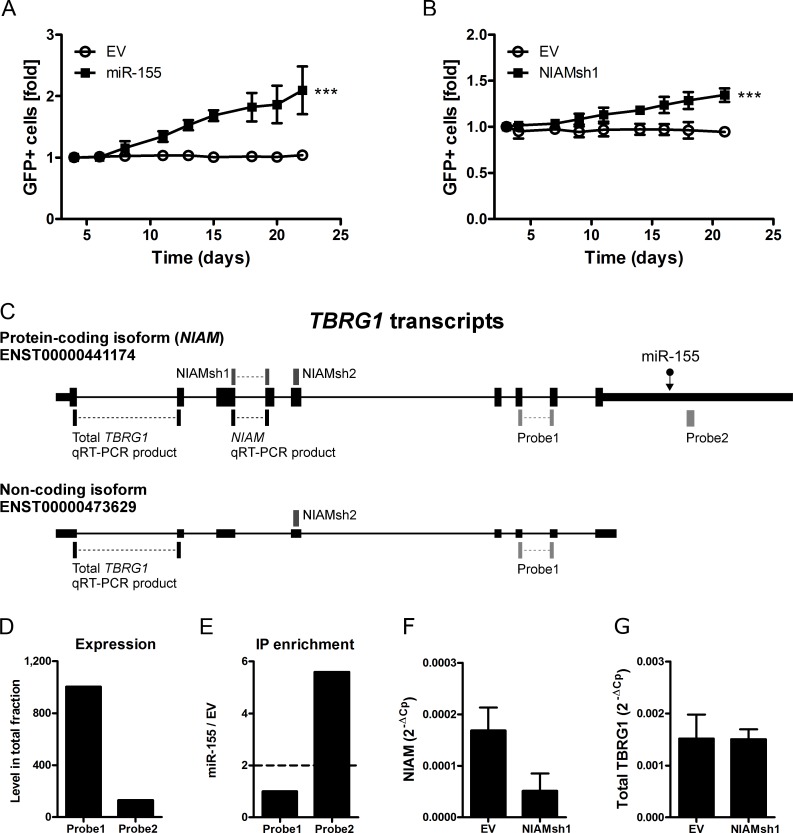
Inhibition of miR-155 target NIAM phenocopies the effect of miR-155 overexpression **A**. Overexpression of miR-155 enhanced growth of ST486 cells resulting in a ∼2 fold increase in the percentage of GFP+ cells on day 22 post-transduction compared to day 4 (mixed model analysis, *** *p* < 0.001). **B**. The NIAM specific shRNA (NIAMsh1) that specifically inhibits the protein-coding isoform (*NIAM*) of the *TBRG1* locus enhanced growth of ST486 cells in the GFP competition assay (mixed model analysis, *** p<0.001). **C**. Schematic overview of the two most abundant *TBRG1* isoforms. The position of the miR-155 binding site (miR-155), the region targeted by the shRNAs against NIAM (NIAMsh), the location of the two probes targeting transcripts of the *TBRG1* locus present on the microarray and the qRT-PCR products specific for *NIAM* and total *TBRG1* are indicated. **D**. The normalized signal intensity of probe 1 (detecting both *TBRG1* isoforms) was much higher than that of probe 2 (*NIAM* only) in ST486 cells. **E**. Probe 2 that specifically detected the *NIAM* transcript was strongly enriched in the targetome of miR-155-ST486 cells, whereas probe 1 that detected both isoforms was not enriched. **F**. QRT-PCR expression analysis confirms that NIAMsh1 can effectively target the *NIAM* transcript but has no impact on the total *TBRG1* transcript level **G**.. The PCR products amplified in panel F and G are indicated in panel C. Expression is relative to ***GAPDH***.

In line with an oncogenic role in B-cell lymphoma, overexpression of miR-155 in ST486 cells caused accelerated growth, resulting in ∼2-fold increase in the percentage of GFP+ cells in 22 days (Figure 2A). Consistent with the mild effects of the miR-155 sponge in the Ago2-IP experiments, no clear effects on cell growth were observed upon overexpression of the miR-155 sponge in HL cell lines L1236 and KM-H2 (data not shown).

To determine whether inhibition of any of the 6 validated miR-155 target genes could reproduce the miR-155 overexpression phenotype we generated two shRNA constructs per gene. For 5 of the 6 targets at least one of the 2 shRNAs showed a 50% reduction at the protein or RNA level (Suppl. Fig. S3A and S3B). For HOMEZ both shRNAs were not effective (data not shown). GFP competition assays using all shRNAs revealed that for only one of the miR-155 target genes, i.e. NIAM, an increased cell growth was observed. Inhibition of NIAM using NIAMsh1 but not NIAMsh2 increased the number of GFP+ cells by ∼50%, approximately half of the effect of miR-155 overexpression (Figure 2B). To explain the discrepancy between the two NIAM shRNAs, we studied the expression pattern of *TBRG1* splice variants. Published RNA-seq data in BL cell line Mutu1 revealed that two isoforms were most abundant, i.e. isoform ENST000004411740 (the protein-coding transcript variant known as *NIAM* which contains a miR-155-binding site) and ENST00000473629 (non-coding *TBRG1* transcript with no miR-155-binding site) (Figure 2C) [14]. The probe on our microarray that detects both isoforms (probe 1) showed signals ∼8 times higher than the probe that specifically detects the miR-155-binding site containing *NIAM* transcript (probe 2) (Figure 2D). However, only the latter probe detecting the *NIAM* transcript was enriched in the IP fraction (Figure 2E). NIAMsh1, that phenocopied the miR-155 overexpression effect, was directed against the *NIAM* transcript and not the non-coding *TBRG1* transcript (Figure 2C). This resulted in >60% downregulation of the *NIAM* transcript while the total *TBRG1* transcript levels were not affected (Figure 2F and 2G). NIAMsh2, which was directed against both isoforms of the *TBRG1* locus, did not show any effect in the GFP competition assay. In line with this, NIAMsh2 did not affect *NIAM* transcript levels (data not shown) possibly due to the much higher abundance of the non-protein-coding transcript. Despite several attempts we were not able to design a second effective shRNA targeting the 137nt exon 4 that is specific to the *NIAM* transcript. Thus, NIAM inhibition phenocopied the growth promoting effect of miR-155 overexpression although this was not confirmed with a second shRNA.

### NIAM expression is inversely associated with miR-155 levels in B-cell lymphoma

**Figure 3:**
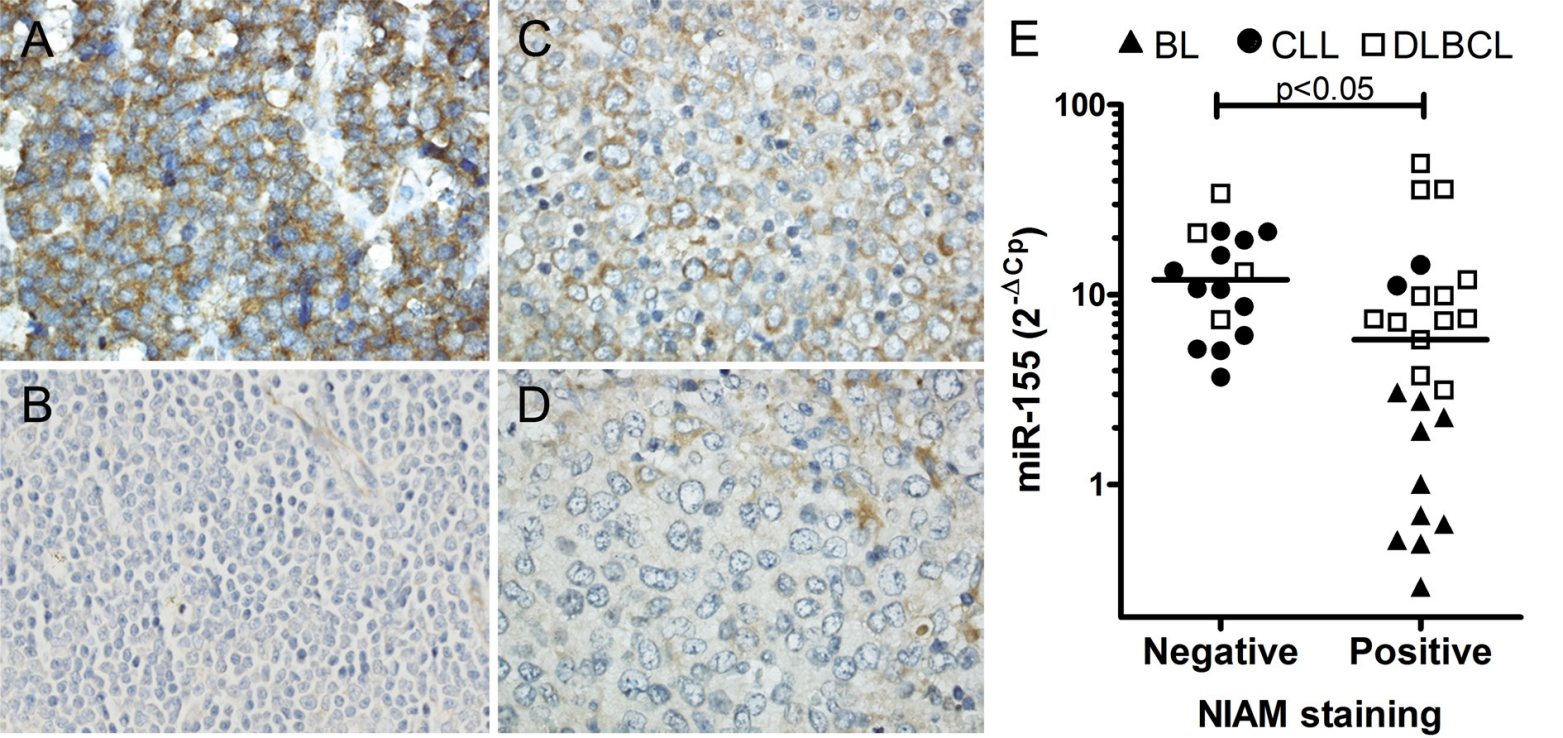
Inverse correlation between NIAM protein expression and miR-155 levels in primary cases of B-cell lymphoma Sections from B-cell lymphoma cases with various levels of miR-155 were stained with anti-NIAM antibody. **A**. BL case positive for NIAM. **B**. CLL case negative for NIAM. **C**. A NIAM-positive and **D**. NIAM-negative DLBCL case. Representative images are shown. Image magnifications 400x. **E**. Significant differences in miR-155 levels were observed between primary B-cell lymphoma cases with or without NIAM protein expression (Mann Whitney U test, * *p* < 0.05). MiR-155 levels were determined by qRT-PCR and normalized to RNU49 levels.

To determine whether NIAM protein expression in primary B-cell lymphoma is inversely associated with the miR-155 expression level we analyzed primary cases of CLL (high miR-155), DLBCL (variable miR-155) and BL (low miR-155) by immunohistochemistry for NIAM and qRT-PCR for miR-155. Two of the 14 CLL cases (14%), 13 out of 17 (76%) DLBCL cases and all 10 BL cases stained positive for NIAM (Figure 3A-3D, Suppl. Fig. S3C for validation of the antibody specificity). Among DLBCL, 75% of the ABC subtype and 78% of the GCB subtype were NIAM-positive. MiR-155 levels were significantly higher in NIAM-negative as compared to NIAM-positive lymphoma cases (*p* < .0145, Mann Whitney U test, Figure 3E). Thus, these data support a miR-155-dependent regulation of NIAM protein expression in primary B-cell lymphoma.

## DISCUSSION

MiR-155 is one of the most studied miRNAs in B-cell lymphoma. Over the past few years several miR-155 target genes were identified, however only a few were shown to be relevant for the pathogenesis of B-cell lymphoma. In this study we identified miR-155 targets in B-cell lymphoma and showed that growth of BL-derived ST486 cells was enhanced upon overexpression of miR-155. Phenotype copy experiments indicated that one of the identified miR-155 targets, i.e. NIAM, is involved in the miR-155-induced enhanced growth. The shRNAs against JARID2, PSIP1, TRIM32 and DET1 did not result in an enhanced cell growth indicating that these genes are unlikely to be involved in the enhanced growth phenotype. However, it might be that JARID2, PSIP1, TRIM32 and DET1 are involved in other miR-155-dependent phenotypes that were not studied.

We identified 54 miR-155 target genes using Ago2-RIP-Chip in ST486 cells with ectopic expression of miR-155. Meier et al. performed Ago2-RIP-Seq in embryonic kidney HEK293 cells with ectopic miR-155 expression and identified 100 miR-155 target genes [15]. In good concordance with our study, their list included 22 of the 54 genes we identified in ST486 cells, including *DET1, TRIM32, HOMEZ* and *JARID2*. These genes likely represent miR-155 targets common to various cell types. *NIAM* was not identified as a miR-155 target in HEK293 cells suggesting that *NIAM* might be a lymphoma-specific target gene.

We were not able to efficiently identify miR-155 target genes using the miR-155-sponge approach in HL cells. MiR-155 sponge transcripts were expressed and efficiently enriched in the Ago2-IP fraction [16], yet miR-155 target genes were only very mildly depleted in the Ago2-IP fraction of miR-155 sponge-transduced HL cells. Although miRNA-sponges can function as very potent miRNA targets that specifically sequester the miRNA to prevent it from binding to endogenous targets it is very well possible that sponge transcript levels in this case were insufficient for complete sequestering of the very highly expressed miR-155 [16-18].

Dorsett et al. showed that low miR-155 levels in normal B cells result in increased expression of the miR-155 target gene Activation-Induced Cytidine Deaminase (*AICDA*) and this enhanced the frequency of *MYC* translocations [19]. Since *MYC* translocations are the hallmark of BL, it might be anticipated that the low miR-155 levels observed in BL are especially crucial at the initiation step of the malignant transformation of germinal center B cells. A recent follow-up study showed that mature B cells isolated from miR-155 deficient mice have higher p53 activity [20]. This was shown to be mediated in part by AICDA and in part by the miR-155 target SOCS1. In BL, the putative undesired activation of p53 via increased expression of the miR-155 target SOCS1 needs to be blocked by direct or indirect inactivation of p53. In line with the proposed oncogenic role of miR-155, in this study we showed that ectopic miR-155 overexpression enhances the growth of BL cell line ST486. However, miR-155 most likely does not function as an oncogene in the pathogenesis of BL, since miR-155 levels in general are low in BL. This is in contrast to other subtypes of B-cell lymphoma that have strongly increased levels of miR-155 and do not depend on AICDA-driven *MYC* translocations, but may benefit from the miR-155-mediated suppression of SOCS1 resulting in reduced p53 activity [6-8].

Inhibition of NIAM expression recapitulated, at least in part, the growth promoting phenotype induced by miR-155 overexpression in ST486 cells. We were not able to quantify the endogenous NIAM levels by Western blotting using the commercial NIAM antibody or a published NIAM antibody [21]. It should be noted that cellular NIAM levels are maintained at relative low levels due to Mdm2-dependent ubiquitination [22]. The inability to detect the endogenous NIAM also prevented a comparison between NIAM protein levels upon miR-155 overexpression and upon shRNA-based NIAM inhibition. Thus, we could not determine whether differences in the effects of miR-155 overexpression and NIAM knockdown are caused by differences in NIAM protein levels or due to other genes simultaneously targeted by miR-155.

We showed that NIAM protein expression inversely correlated with miR-155 levels in primary B-cell lymphoma. NIAM staining was predominantly cytoplasmic. This is in contrast to the results reported by Reed et al., who showed that NIAM is localized in the nucleus and bound to chromatin [23]. Nonetheless and similar to our study, NIAM was clearly localized in the cytoplasm of 2 pancreatic cell lines [21]. These different staining patterns indicate that NIAM can be both nuclear and cytoplasmic. Microarray studies indicated that *NIAM* transcript levels are lowered in various types of cancer, including B-cell lymphoma [22]. The tumor suppressive function of NIAM was confirmed in a recent study that showed that NIAM-deficient mice were predisposed to develop proliferative lesions, including early stage B-cell lymphoma [24]. Moreover, NIAM was shown to act as a tumor suppressor protein linked to the p53 pathway in several ways [22, 23]. It stimulated p53 activity by interacting with p53 regulators such as Mdm2 and Tip60 [23]. Exogenous NIAM stabilized p53 by binding to Mdm2 and preventing Mdm2-mediated p53 ubiquitylation, and association of NIAM with Tip60 increased p53 transactivation of the p21 promoter. In addition, exogenous NIAM was shown to inhibit cell proliferation independent of p53 [25]. It would be interesting to study whether the NIAM-mediated effect of miR-155 depends on decreased p53 activity.

In conclusion, we identified NIAM as a novel miR-155 target gene in B-cell lymphoma. Induction of miR-155 enhances growth of ST486 BL cells and our data indicate that this is at least in part caused by inhibition of NIAM. NIAM expression inversely correlated with miR-155 levels in primary B-cell lymphoma. Our data, together with the recent observation that NIAM-deficient mice are predisposed to malignant transformation suggest that NIAM is a crucial target for the oncogenic effects of miR-155 in B-cell lymphoma.

## MATERIALS AND METHODS

### Tissue samples

Individual diagnosis of the 10 BL, 14 CLL and 17 DLBCL samples were reviewed by an experienced hematopathologist for consistent morphology and immunophenotype according to the 2008 WHO classification [26]. Pediatric BL cases presented with an abdominal mass and were CD10+, BCL2- and MYC-breakpoint+. CLL cases were nodal and CD5+, CD23+, cyclinD1- with variable ZAP70 expression. Diffuse large B-cell lymphoma (DLBCL) cases were classified into ABC (8) or GCB (9) subtypes according to the Hans algorithm [27]. All protocols for obtaining human tissue samples were performed in accordance to the guidelines from the Institutional review board or Medical Ethical committee of the University Medical Center Groningen.

### Cell lines

ST486, L1236 and KM-H2 cell lines were cultured as previously described [28, 29]. Cell lines were purchased from ATCC (ST486) or DSMZ (L1236 and KM-H2). Phoenix-ampho cells were cultured in DMEM supplemented with 10% fetal calf serum.

### DNA constructs and viral transductions

To overexpress miRNA-155, the pre-miRNA with flanking sequences was amplified from genomic DNA using primers listed in Table S6. Insert was cloned into the retroviral MXW-PGK-IRES-GFP vector [30] using XhoI and EcoRI. To validate miRNA overexpression, GFP+ cells were sorted ∼2 weeks after transduction using a MoFlo sorter (Dako cytomation). We inhibited miR-155 using a retrovirally expressed miR-155 sponge with 14 binding sites (Table S6) [16]. The 3′UTR of *DET1, TRIM32, JARID2, PSIP1, NIAM* and the ∼300nt coding sequence fragment of *HOMEZ* containing the potential miR-155-binding sites were amplified from genomic DNA using primers listed in Table S6 and cloned into the psiCHECK2 vector (Promega, Madison, WI). To inhibit miR-155 target genes, shRNA oligos were designed (Table S6) and cloned into the retroviral pMDH1-PGK-GFP 2.0 vector [30]. The sequence of NIAMsh1 was based on a previous study [22]. Retroviral transductions were performed as previously described [28].

### Quantitative RT-PCR

RNA isolation and miRNA-specific cDNA synthesis and qPCR were performed as previously described [31, 32]. MiRNA levels were normalized to RNU48 or RNU49 levels. For quantification of *NIAM*, total *TBRG1, DET1, TRIM32, HOMEZ, PSIP1, JARID2*, cDNA was synthesized using random primers, dNTP mix and the Superscript II Reverse Transcriptase Kit (Life Technologies Europe BV, Bleiswijk, NL) according to manufacturer's instructions. The qPCR reactions for *NIAM* and total *TBRG1* were performed as previously described [28], *GAPDH* was used as a reference gene, primers are listed in Table S6. For *DET1, TRIM32, PSIP1* and *JARID2*, the qPCR reactions were performed using qPCR MasterMix Plus (Eurogentec, Liege, Belgium) and either Taqman Gene expression assays (Hs00894490_m1 for *DET1*, Hs00705875_s1 for *TRIM32*, Hs01045711_g1 for *PSIP1* and Hs01004460_m1 for *JARID2*, all Applied Biosystems) or designed primers and probe for detection of *HPRT* as decribed previously [33].

### Ago2-RIP-chip procedure

Immunoprecipitation of Ago2-containing RISC complexes was performed as described previously [34]. Briefly, cleared lysates of 20-30 million cells were incubated with protein G Sepharose beads (GE Healthcare) coated with anti-Ago2 antibody (Clone 2E12-1C9, Abnova, Taiwan) at 4&deg;C overnight. Anti-IgG antibody was used as a negative control (Millipore BV, Amsterdam, The Netherlands). RNA was isolated for microarray and qRT-PCR analysis and protein lysates were prepared for Western blot. Western blot for Ago2 was performed as described previously [34]. RNA from the total and Ago2-IP fractions was analyzed using 44k Human Whole Genome Oligo microarrays (Agilent, Santa Clara, USA). Labeling and hybridization were performed with 100ng of total RNA using the two-color Quick Amp Labeling Kit (ST486) or Low Input Quick Amp Labeling Kit (L1236 and KM-H2) and Cyanine (Cy) 3 and 5 CTP Dye Packs according to the manufacturer's protocol (Agilent). The microarray data have been deposited in NCBI's Gene Expression Omnibus [35] and are accessible through GEO Series accession number GSE70939. Data were analyzed using GeneSpring GX version 12.5 (Agilent). Quantile normalization of the signals was performed. Probes not detected in more than half of the samples and that were inconsistent (more than 2 fold different) in Cy3 and Cy5 replicates of the same sample were filtered out. The averaged signals for Cy3 and Cy5 replicates were used to calculate the IP/T ratio for each sample.

### Gene set enrichment analysis

To determine which genes sets are significantly enriched in the Ago2-IP in comparison to the total fraction we performed Gene Set Enrichment Analysis using The Molecular Signatures Database (GSEA; http://www.broad.mit.edu/gsea) [36]. If more than one probe was assigned for a certain gene, we selected the probe with the highest IP fold enrichment.

### Transfection and luciferase assay

Luciferase assays were performed using the Promega Dual-Luciferase Reporter Assay System (Promega, Madison, WI) as described previously [37]. Briefly, two million ST486 cells were transfected with 4&micro;g of each psiCHECK2 construct and co-transfected with 100nM miR-155 precursor or negative control #1 (both Ambion) using an Amaxa nucleofector device, program A23 (Amaxa, Gaithersburg, MD). Transfections were performed in triplicate and cell lysates were made 24h after transfection. Renilla to Firefly (RL/FL) luciferase ratios were calculated and compared to negative control (set at 100%). Significance was calculated using the t-test.

### Western blot

Western blot for DET1 was performed as previously described [38]. Immunoblots were incubated with mouse anti-DET1 antibody (clone 3G5, Genentech, San Francisco, CA) at a concentration of 1 &micro;g/ml in 5% milk in Tris-buffered saline with Tween-20 (TBST) overnight at 4&ordm;C. Mouse anti-GAPDH antibody (clone 0411, 1:20,000, Santa Cruz, CA, USA) was used as a control. Western Blot for NIAM was performed using a rabbit polyclonal anti-NIAM antibody (18951-1-AP, Proteintech, Chicago, IL, USA) diluted 1:500 in 5% milk in TBST overnight at 4&ordm;C.

### GFP competition assay

GFP expression was measured on a FACS Calibur flow cytometer (BD PharMingen) at day 3 or 4 post-transduction and monitored for three weeks tri-weekly. The percentage of GFP+ cells was analyzed using FlowJo software (version 7.6, Treestar, Ashland, OR); the value at day 3 or 4 was set to 1 and the fold difference per measurement was calculated. To determine whether cells with increased miR-155 or decreased TBRG1 levels grow significantly different from cells transduced with control vectors, we performed mixed model analysis on the relative percentages with the interaction between transduced cell type and day of measurement as fixed effects and day of measurements and the biological replicate measurements as within-subject variables in SPSS (version 22). Since the relative percentages were 1 on day 3 or 4 by definition, we analyzed the relative percentage −1 and did not include the intercept and the main effects of cell type and day of measurement in the model.

### NIAM immunohistochemistry

Sections of BL, CLL and DLBCL cases were cut (3&micro;m) and staining was performed using a rabbit polyclonal anti-NIAM antibody (18951-1-AP, 1:25, Proteintech) in combination with microwave-citrate buffer (pH 6.0) antigen retrieval. Staining was visualized using a peroxidase coupled goat anti-rabbit secondary antibody (1:100), a peroxidase-coupled rabbit anti-goat tertiary antibody (1:100, both DAKO, Glostrup, Denmark), and 3,3-diaminobenzidine tetrachloride.

## SUPPLEMENTARY FIGURES AND TABLES


