# Supplementary material for: Inhibition of the miR-155 target NIAM phenocopies the growth promoting effect of miR-155 in B-cell lymphoma
Source: Oncotarget. 2015 Oct 19;7(3):2391–400. doi: 10.18632/oncotarget.6165 (PMC4823043; doi:10.18632/oncotarget.6165)
Supplement: Supplementary file 1 [file oncotarget-07-2391-s001.pdf]

Inhibition of the miR-155 target NIAM phenocopies the growth promoting effect of miR-155 in B-cell lymphoma

Supplementary Material

Supplementary Table S1: Similar numbers of probes were identified in the miRNA-targetome sets per cell line

	ST486 (n=14,453)		***L1236 (n=22,282)***		***KM-H2 (n=16,065)***	
**Enrichment**	**EV**	**miR-155**	**EV**	**miR-155 sponge**	**EV**	**miR-155 sponge**
IP/T≥2	1,804 (12.5%)	1,833 (12.7%)	3,928 (17.6%)	3,945 (17.7%)	2,432 (15.1%)	2,577 (16.0%)
IP/T≥4	664 (4.6%)	701 (4.9%)	1,332 (6.0%)	1,276 (5.7%)	1,024 (6.4%)	1,090 (6.8 %)
IP/T≥8	239 (1.7%)	226 (1.6%)	382 (1.7%)	356 (1.6%)	386 (2.4%)	422 (2.6%)

IP = immunoprecipitated fraction, T = total fraction, EV = empty vector

Supplementary Table S2: **MiR-155 target genes in miR-155-ST486 cells identified with Ago2-RIP-Chip**

Gene	RefSeq	ProbeName	miR-155 IP/T	EV IP/T	miR-155/ EV	TS*	# 8-mer[†] in 3'UTR	# 6-mer[‡] in 3'UTR
DET1	NM_017996	A_23_P26184	17.5	1.4	12.6	+	1	3
TBRG1 (NIAM)	NM_032811	A_23_P98463	11.6	2.2	5.4	+	1	2
TRIM32	NM_012210	A_23_P112311	2.1	0.4	5.2	+	1	1
HOMEZ	NM_020834	A_23_P76829	5.1	1.1	4.7		0[§]	0[§]
PSIP1	NM_021144	A_23_P256384	9.8	2.4	4.1	+	1	2
C14orf159	BC009182	A_24_P58177	2.0	0.6	3.7		0	1
CCDC126	NM_138771	A_23_P168592	4.0	1.1	3.7		1	3
MAX	NM_145114	A_23_P436138	2.1	0.6	3.7		0	0
PSKH1	NM_006742	A_23_P390596	11.7	3.3	3.5	+	1	1
BRWD1	NM_018963	A_24_P190541	3.5	1.1	3.3	+	0	2
ZNF578	NM_001099694	A_23_P339601	5.0	1.5	3.3		1	1
IER5	NM_016545	A_23_P86330	3.4	1.1	3.1		0	1
TCF4	NM_003199	A_23_P27332	3.1	1.0	3.1	+	2	4
SAR1A	NM_020150	A_23_P127175	4.6	1.5	3.0		0	0
KLHL5	NM_015990	A_23_P121527	3.9	1.3	3.0		1	3
TBC1D14	NM_020773	A_24_P120352	2.3	0.8	3.0		1	1
JARID2	NM_004973	A_23_P214876	6.8	2.2	3.0	+	2	2
PRDM15	AY063456	A_32_P145989	2.8	1.0	3.0		1	2
ZNF845	NM_138374	A_32_P207428	6.2	2.1	2.9		1	2
USPL1	NM_005800	A_24_P338757	6.4	2.2	2.9		0	1
DPY19L1	NM_015283	A_23_P358628	3.4	1.2	2.8		1	3
ARRDC2	NM_001025604	A_23_P130965	2.1	0.7	2.8		0	1
FAM119A	NM_145280	A_23_P209337	2.6	1.0	2.7		0	0
PHKB	NM_001031835	A_23_P206532	2.2	0.8	2.7		0	1
TAB2	NM_015093	A_23_P19702	7.1	2.7	2.6	+	1	2
ERI2	NM_080663	A_23_P129717	6.6	2.5	2.6		0	0
PICALM	NM_007166	A_23_P147995	2.2	0.9	2.6		0	4
ZFP36	NM_003407	A_23_P39237	19.4	7.6	2.6		0	1
VAMP3	NM_004781	A_24_P370887	20.2	7.9	2.5		0	3
CENPI	NM_006733	A_23_P252292	2.1	0.8	2.5		0	0
BACH1	NM_206866	A_23_P211047	14.5	5.8	2.5	+	1	3
RHEB	NM_005614	A_23_P134247	3.8	1.5	2.5	+	0	1
PHC2	NM_198040	A_23_P423864	2.6	1.1	2.4	+	1	1
ZNF320	NM_207333	A_32_P540407	6.1	2.5	2.4		1	2
PLEKHB2	NM_001100623	A_24_P873414	4.6	1.9	2.4		1	1
C5orf15	NM_020199	A_23_P81650	3.2	1.3	2.4		0	0
ARFIP1	NM_001025595	A_24_P166094	2.3	1.0	2.4		0	1
CLUAP1	NM_024793	A_23_P77714	2.6	1.1	2.3		1	1
RNF26	NM_032015	A_23_P64630	2.2	1.0	2.3		1	1
PPA2	NM_176869	A_24_P214625	3.0	1.3	2.2		0	0
PANK1	NM_148977	A_23_P127054	3.7	1.7	2.2	+	0	1
GALT	NM_000155	A_24_P12865	2.4	1.1	2.1		1	1
TPD52	NM_001025252	A_23_P216257	2.6	1.2	2.1		0	1
ZNF137	NR_023311	A_23_P208238	3.1	1.5	2.1		0	0
CD58	NM_001779	A_23_P138308	3.8	1.9	2.1		0	2
CSRP2	NM_001321	A_23_P44724	10.8	5.2	2.0		1	2
LIN9	NM_173083	A_23_P301995	2.2	1.1	2.0		0	1
DCTN6	NM_006571	A_23_P43049	8.8	4.3	2.0		0	0
FGF7	NM_002009	A_23_P14612	10.7	5.3	2.0	+	1	2
KIAA1715	CR936742	A_32_P127248	2.6	1.3	2.0	+	0	2
MARK2	NM_004954	A_24_P914495	3.3	1.6	2.0	+	0	1
CSNK1G2	NM_001319	A_24_P99963	3.2	1.6	2.0	+	1	1
MFSD5	NM_032889	A_23_P72850	5.0	2.5	2.0		0	0
LNX2	NM_153371	A_23_P402287	3.4	1.7	2.0	+	1	1

* TS = miR-155 target predicted by TargetScan 6.2, [†]8-mer sequence = AGCATTAA, [‡]6-mer sequence = GCATTA, [§]8-mer and 6-mer *in CDS*

Supplementary Table S3: 20 most enriched gene sets in EV-ST486 and miR-155-ST486 cells

Gene set	Position in GSEA	
	EV	miR-155
GCACTTT,MIR-17-5P,MIR-20A,MIR-106A,MIR-106B,MIR-20B,MIR-519D	1	1
ACACTAC,MIR-142-3P	2	3
AGCACTT,MIR-93,MIR-302A,MIR-302B,MIR-302C,MIR-302D,MIR-372,MIR-373,MIR-520E,MIR-520A,MIR-526B,MIR-520B,MIR-520C,MIR-520D	3	2
CTACTGT,MIR-199A	4	8
GTGCAAT,MIR-25,MIR-32,MIR-92,MIR-363,MIR-367	5	4
TGAATGT,MIR-181A,MIR-181B,MIR-181C,MIR-181D	6	6
TTTGCAC,MIR-19A,MIR-19B	7	7
AAGCACT,MIR-520F	8	10
ACTTTAT,MIR-142-5P	9	11
NAGASHIMA_NRG1_SIGNALING_UP	10	9
TGCACTT,MIR-519C,MIR-519B,MIR-519A	11	5
KIM_WT1_TARGETS_UP	12	14
TTGCCAA,MIR-182	13	41
BONCI_TARGETS_OF_MIR15A_AND_MIR16_1	14	24
TGCACTG,MIR-148A,MIR-152,MIR-148B	15	12
TGTTTAC,MIR-30A-5P,MIR-30C,MIR-30D,MIR-30B,MIR-30E-5P	16	20
PICCALUGA_ANGIOIMMUNOBLASTIC_LYMPHOMA_DN	17	18
ATAAGCT,MIR-21	18	32
TACTTGA,MIR-26A,MIR-26B	19	15
AMIT_EGF_RESPONSE_40_HELA	20	25
AGCATTA,MIR-155	**46**	**13**
NAGASHIMA_EGF_SIGNALING_UP	35	16
ACATATC,MIR-190	23	17
TONKS_TARGETS_OF_RUNX1_RUNX1T1_FUSION_HSC_UP	30	19

Supplementary Table S4: 20 most enriched gene sets in L1236 cells with EV and miR-155 sponge

Gene set	Position in GSEA	
	EV	miR-155 sponge
STK33_UP	1	1
ACACTAC,MIR-142-3P	2	2
STK33_NOMO_UP	3	3
IVANOVSKA_MIR106B_TARGETS	4	4
CHR19P12	5	6
GCACTTT,MIR-17-5P,MIR-20A,MIR-106A,MIR-106B,MIR-20B,MIR-519D	6	5
STK33_SKM_UP	7	7
AGCATTA,MIR-155	**8**	**9**
GSE29617_CTRL_VS_DAY7_TIV_FLU_VACCINE_PBMC_2008_UP	9	10
AGCACTT,MIR-93,MIR-302A,MIR-302B,MIR-302C,MIR-302D,MIR-372,MIR-373,MIR-520E,MIR-520A,MIR-526B,MIR-520B,MIR-520C,MIR-520D	10	8
GTGCAAT,MIR-25,MIR-32,MIR-92,MIR-363,MIR-367	11	11
GSE14769_UNSTIM_VS_40MIN_LPS_BMDM_DN	12	12
GSE29617_CTRL_VS_TIV_FLU_VACCINE_PBMC_2008_UP	13	16
LINSLEY_MIR16_TARGETS	14	15
TGCACTT,MIR-519C,MIR-519B,MIR-519A	15	13
ZWANG_CLASS_3_TRANSIENTLY_INDUCED_BY_EGF	16	34
ATACCTC,MIR-202	17	26
ACTTTAT,MIR-142-5P	18	17
GSE14769_UNSTIM_VS_120MIN_LPS_BMDM_DN	19	20
TTTGCAC,MIR-19A,MIR-19B	20	14
GSE36476_CTRL_VS_TSST_ACT_72H_MEMORY_CD4_TCELL_YOUNG_UP	21	18
GSE9988_ANTI_TREM1_AND_LPS_VS_CTRL_TREATED_MONOCYTES_UP	25	19

Supplementary Table S5: 20 most enriched gene sets in KM-H2 cells with EV and miR-155 sponge

Gene set	Position in GSEA	
	EV	miR-155 sponge
IVANOVSKA_MIR106B_TARGETS	1	1
CHR19P12	2	3
STK33_UP	3	5
STK33_NOMO_UP	4	4
GCACTTT,MIR-17-5P,MIR-20A,MIR-106A,MIR-106B,MIR-20B,MIR-519D	5	2
ACACTAC,MIR-142-3P	6	7
GTGCAAT,MIR-25,MIR-32,MIR-92,MIR-363,MIR-367	7	6
AGCACTT,MIR-93,MIR-302A,MIR-302B,MIR-302C,MIR-302D,MIR-372,MIR-373,MIR-520E,MIR-520A,MIR-526B,MIR-520B,MIR-520C,MIR-520D	8	10
ACTACCT,MIR-196A,MIR-196B	9	11
STK33_SKM_UP	10	18
ATACCTC,MIR-202	11	8
AMIT_EGF_RESPONSE_40_HELA	12	9
ZWANG_CLASS_3_TRANSIENTLY_INDUCED_BY_EGF	13	12
GARGALOVIC_RESPONSE_TO_OXIDIZED_PHOSPHOLIPIDS_TURQUOISE_UP	14	15
CTACCTC,LET-7A,LET-7B,LET-7C,LET-7D,LET-7E,LET-7F,MIR-98,LET-7G,LET-7I	15	13
TGCACTT,MIR-519C,MIR-519B,MIR-519A	16	14
LINSLEY_MIR16_TARGETS	17	21
PIGF_UP.V1_UP	18	19
GSE29617_CTRL_VS_DAY7_TIV_FLU_VACCINE_PBMC_2008_UP	19	22
GSE17974_CTRL_VS_ACT_IL4_AND_ANTI_IL12_0.5H_CD4_TCELL_DN	20	40
TTTGCAC,MIR-19A,MIR-19B	23	16
ATGCTGC,MIR-103,MIR-107	26	17
ACTTTAT,MIR-142-5P	27	20
AGCATTA,MIR-155	**25**	**24**

Supplementary Table S6: Oligonucleotide sequences

Name	Sequence (5'-3')
Luciferase constructs	
DET1 3'UTR-F	GAGCTCGTGCCTCACCAGAGCCAGAT
DET1 3'UTR-R	GCGGCCGCCACTTAGTTCTCCCAGGAACAG
NIAM 3'UTR-F	GAGCTCACAAGAAGGGATCAGATGCCACATCG
NIAM 3'UTR-R	GCGGCCGCGTGCCACCACGCCTGGCTAATTT
TRIM32 3'UTR-F	GAGCTCGAGAAATTATCAGTTTCTTCTGC
TRIM32 3'UTR-R	GCGGCCGCGTTCAACATCATTTTAATGACC
HOMEZ CDS-F	CGTGCAACGAGCTCATGGCATAGGTACTGCTTCC
HOMEZ CDS-R	CTTAGCACGCGGCCGCGAGTTATGCCGTAGCCCTTG
PSIP1 3'UTR-F	GAGCTCTTGGGCTCAAAGCATTAATC
PSIP1 3'UTR-R	GCGGCCGCTTTGGTTACAGTTTCATTCTT
JARID2 3'UTR-F	GAGCTCAACGCCCGTGGTCGATTTAT
JARID2 3'UTR-R	GCGGCCGCTATTATTAAACCTTGTAGTACAAAC
miR-155 overexpression construct	
miR-155-F	CTTGGCTAGCTCGAGTGTCACTCCAGCTTTATAACC
miR-155-R	CATGGAATTCACAGATTGAAAAATGATAAAGCC
miR-155 sponge construct (14xMBS)	CTCGAGCTGGTTAACGACGGGTCC**ACCCCTATGGAATTAGCATTAA**AATT**ACCCCTATGGAATTAGCATTAA**GTCC**ACCCCTATGGAATTAGCATTAA**AATT**ACCCCTATGGAATTAGCATTAA**GTCC**ACCCCTATGGAATTAGCATTAA**AATT**ACCCCTATGGAATTAGCATTAA**GTCC**ACCCCTATGGAATTAGCATTAA**AATT**ACCCCTATGGAATTAGCATTAA**GTCC**ACCCCTATGGAATTAGCATTAA**AATT**ACCCCTATGGAATTAGCATTAA**GTCC**ACCCCTATGGAATTAGCATTAA**AATT**ACCCCTATGGAATTAGCATTAA**GTCC**ACCCCTATGGAATTAGCATTAA**AATT**ACCCCTATGGAATTAGCATTAA**GTCCCGACGTTTAAACGACGAATTC
shRNA sequences	
DET1sh1	CTCGAGAACGTTGAAAAGCCTCCTTGTCTCGAGACAAGGAGGCTTTTCAACGTTTTTTTGAATTC
DET1sh2	CTCGAGAAGACTATTCCCTCCATATCACTCGAGTGATATGGAGGGAATAGTCTTTTTTTGAATTC
NIAMsh1	CTCGAGACTGGAAGTTCTGAAGAAACTCGAGTTTCTTCAGAACTTCCAGTTTTTTGAATTC
NIAMsh2	CTCGAGCCAGACCAGAAGTGTCTATATCTCGAGATATAGACACTTCTGGTCTGGTTTTTGAATTC
TRIM32sh1	CTCGAGATAACTCCCTCAAGGTATATACTCGAGTATATACCTTGAGGGAGTTATTTTTTGAATTC
TRIM32sh2	CTCGAGGCCACTTCTTCTCGGAGAATGCTCGAGCATTCTCCGAGAAGAAGTGGCTTTTTGAATTC
HOMEZsh1	CTCGAGAGGCACCATGCCTCCTAATAACTCGAGTTATTAGGAGGCATGGTGCCTTTTTTGAATTC
HOMEZsh2	CTCGAGTACCTCGGCCTGAGATCATTCCTCGAGGAATGATCTCAGGCCGAGGTATTTTTGAATTC
PSIP1sh1	CTCGAGGCAGCAACTAAACAATCAAATCTCGAGATTTGATTGTTTAGTTGCTGCTTTTTGAATTC
JARID2sh1	CTCGAGGAAACAGGTTTCTAAGGTAAACTCGAGTTTACCTTAGAAACCTGTTTCTTTTTGAATTC
JARID2sh2	CTCGAGGCCCAACAGCATGGTGTATTTCTCGAGAAATACACCATGCTGTTGGGCTTTTTGAATTC
qRT-PCR primers	
NIAM-F	ACAAACTGGAAGTTCTGAAG
NIAM-R	AATGGGCTGAACCAGCTTGC
Total TBRG1-F	GCGGCTGCGCAAAGC
Total TBRG1-R	TCATCACAAATAGCAGCATTTT
GAPDH-F	CCACATCGCTCAGACACCAT
GAPDH-R	GCGCCCAATACGACCAAAT

F = forward primer, R = reverse primer, MBS = miRNA binding site, sh=short hairpin, Restriction sites in sequences are underlined (GAGCTC = SstI; GCGGCCGC = NotI; CTCGAG = XhoI; GAATTC = EcoRI). MiR-155 sponge consists of repeats of miRNA binding sites in bold followed by stuffer sequences in normal font.

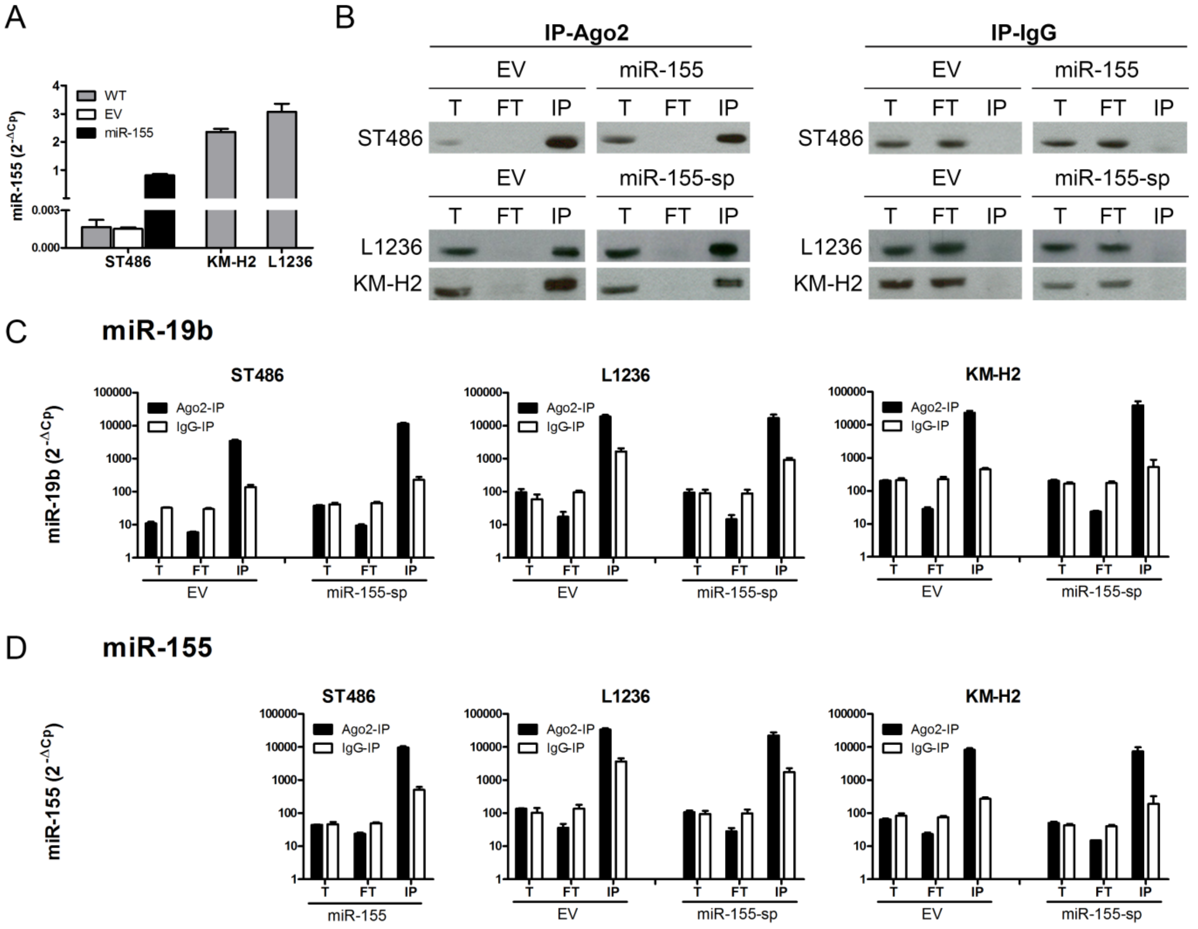

Supplementary Figure S1: Quality control experiments for the Ago2-IP in ST486, L1236 and KM-H2 cells. (A) Overexpression of miR-155 in ST486 cells was confirmed by qRT-PCR in GFP+ ST486 cells. Overexpression levels were in the same range as endogenous miR-155 levels in KM-H2 and L1236 cells. (B) Analysis of the Ago2-immunoprecipitation (IP) by Western blotting for Ago2 protein in total (T), flowthrough (FT) and IP fractions. Ago2 was pulled down using an anti-Ago2 antibody but not when an IgG1 negative control antibody was used in ST486 transduced with empty vector (EV) or miR-155 or in L1236 and KM-H2 transduced with EV or miR-155 sponge (miR-155-sp). (C) As an additional quality control we analyzed the levels of a high-expressed miRNA, i.e. miR-19b, in all 3 cell lines. This miRNA is strongly enriched in all the Ago2-IP fractions but not in the IgG1-IP negative control fractions. (D) MiR-155 was strongly enriched in the Ago2-IP fractions but not in the IgG1-IP negative control fractions. Next to the HL cell lines L1236 and KM-H2, miR-155 levels could only be measured in miR-155-transduced ST486 cells, as the levels in EV-transduced ST486 cells were below the detection limit. MiRNA levels were determined by qRT-PCR relative to RNU48 levels.

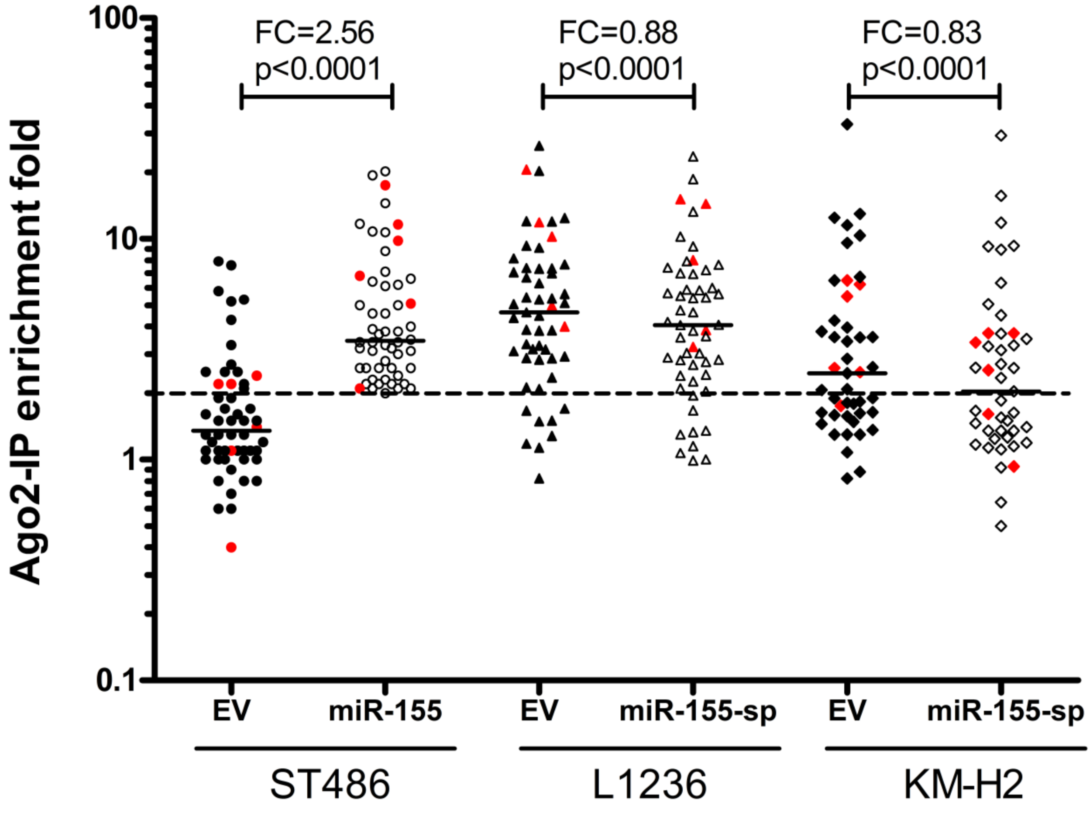

Supplementary Figure S2: Behavior of the miR-155-ST486 target genes in the Ago2-IP experiments. Shown are the Ago2-IP fold enrichments (IP/T ratio) for the 54 miR-155 target genes identified in ST486 cells that were at least 2-fold enriched in ST486 cells upon miR-155 overexpression compared to EV-transduced cells. The majority of these genes are also enriched in the EV-transduced HL cell lines L1236 and KM-H2 (both have high levels of miR-155). Upon overexpression of the miR-155 sponge (miR-155-sp) the fold enrichments of the target genes decrease only mildly (average fold-change (FC) upon sponge overexpression is 0.88 and 0.83 for L1236 and KM-H2 respectively. For each cell line the median fold enrichment is indicated and the 6 validated genes (see Fig. 1B) are highlighted in red. P values were calculated with Wilcoxon matched pairs test.

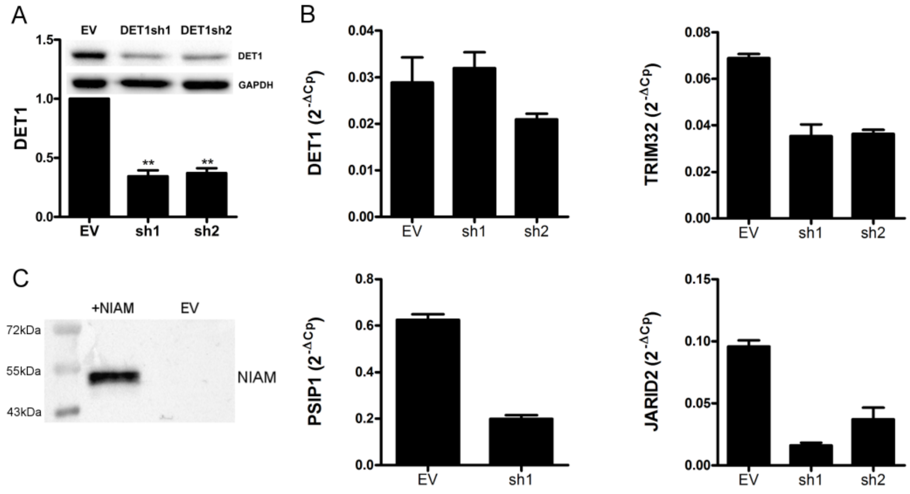

Supplementary Figure S3: Validation of the effectiveness of the shRNAs against the six selected miR-155 target genes and the specificity of anti-NIAM antibody. For all 6 selected miR-155 target genes shRNAs were generated and tested for effectiveness. (A) Validation of the two DET1 shRNAs (DET1sh) at the protein level. Both shRNAs showed a > 60% reduction in DET1 protein levels in ST486 cells. A representative example and the quantification of 2 Western blot experiments are shown. (B) Validation of the shRNAs (sh) at the RNA level. For *DET1* only one of the 2 shRNAs showed a mild effect at the RNA level. For *TRIM32, JARID2* and *PSIP1* all shRNAs showed at least a 50% reduction at the transcript levels. Note that for *PSIP1* only 1 shRNA was available. Results for the *NIAM* shRNAs are presented in figure 2. For *HOMEZ* both shRNAs were not effective (data not shown). Transcript levels were determined by qRT-PCR and normalized to *HPRT*. (C) To ensure that the anti-NIAM antibody used for immunohistochemistry detects NIAM we overexpressed NIAM (+NIAM) in ST486 cells. This resulted in a clear and specific band at the expected height upon NIAM overexpression (50-55 kDa). No band was observed in the empty vector (EV)-transduced cells (endogenous levels too low to detect). Similar results were obtained using a previously published antibody (clone 11E12, a kind gift from Dawn Quelle, data not shown).
